# Altered collective mitochondrial dynamics in the Arabidopsis *msh1* mutant compromising organelle DNA maintenance

**DOI:** 10.1093/jxb/erac250

**Published:** 2022-06-05

**Authors:** Joanna M Chustecki, Ross D Etherington, Daniel J Gibbs, Iain G Johnston

**Affiliations:** School of Biosciences, University of Birmingham, Birmingham, UK; School of Biosciences, University of Birmingham, Birmingham, UK; School of Biosciences, University of Birmingham, Birmingham, UK; Department of Mathematics, University of Bergen, Realfagbygget, Bergen, Norway; Computational Biology Unit, University of Bergen, Høyteknologisenteret i Bergen, Bergen, Norway; University of Nebraska-Lincoln, USA

**Keywords:** *Arabidopsis thaliana*, mitochondrial dynamics, *msh1*, social networks, time-lapse microscopy

## Abstract

Mitochondria form highly dynamic populations in the cells of plants (and almost all eukaryotes). The characteristics and benefits of this collective behaviour, and how it is influenced by nuclear features, remain to be fully elucidated. Here, we use a recently developed quantitative approach to reveal and analyse the physical and collective ‘social’ dynamics of mitochondria in an Arabidopsis *msh1* mutant where the organelle DNA maintenance machinery is compromised. We use a newly created line combining the *msh1* mutant with mitochondrially targeted green fluorescent protein (GFP), and characterize mitochondrial dynamics with a combination of single-cell time-lapse microscopy, computational tracking, and network analysis. The collective physical behaviour of *msh1* mitochondria is altered from that of the wild type in several ways: mitochondria become less evenly spread, and networks of inter-mitochondrial encounters become more connected, with greater potential efficiency for inter-organelle exchange—reflecting a potential compensatory mechanism for the genetic challenge to the mitochondrial DNA population, supporting more inter-organelle exchange. We find that these changes are similar to those observed in *friendly*, where mitochondrial dynamics are altered by a physical perturbation, suggesting that this shift to higher connectivity may reflect a general response to mitochondrial challenges, where physical dynamics of mitochondria may be altered to control the genetic structure of the mtDNA population.

## Teaser

Why do plant mitochondria move? We perturb organelle DNA maintenance via MSH1 and show that collective mitochondrial dynamics are altered to aid more potential exchange between organelles, supporting a link between physical and genetic mitochondrial dynamics.

## Introduction

Mitochondria are key bioenergetic compartments of the eukaryotic cell. Within plant cells, hundreds of mitochondria exist, largely as individual organelles—contrasting with the reticulated network form often seen in yeast and mammalian cells (Logan, 2006b; Johnston, 2019). These cellular populations are highly dynamic (Logan, 2010), interacting with each other and with other organelles (Islam *et al.*, 2009; Jaipargas *et al.*, 2015; Shai *et al.*, 2016; Barton *et al.*, 2018; Krupinska *et al.*, 2020; Chustecki *et al.*, 2021).

Housed within these organelles, mitochondrial DNA (mtDNA) encodes essential information for the mitochondrial machinery. In plant cells, again contrasting with other kingdoms, different mitochondria contain different subsets of the full mtDNA genome (Preuten *et al.*, 2010). Many mitochondria may contain no mtDNA at all, while some may contain the full genome (57 genes across 366 kb in Arabidopsis), and others may contain a subgenomic molecule containing some but not all mtDNA genes (Arimura *et al.*, 2004; Gualberto *et al.*, 2014; Kozik *et al.*, 2019). Processes of mtDNA exchange and recombination are essential to maintain this diverse structure (Bellaoui *et al.*, 1998; Arrieta-Montiel *et al.*, 2009; Davila *et al.*, 2011; Gualberto and Newton, 2017), with mtDNA sharing through the population of mitochondria constituting a ‘discontinuous whole’ (Logan, 2006a).

Such sharing and recombination is inherently shaped and limited by the physical behaviour of organelles in the cell (Belliard *et al.*, 1979; Lonsdale *et al.*, 1988; Gualberto and Newton, 2017; Aryaman *et al.*, 2019; Johnston, 2019; Rose, 2021). In order for this sharing to occur, mitochondria must physically meet and exchange contents—so the genetic structure of the mtDNA population is inherently controlled by the physical dynamics of the mitochondrial compartments.

Recent work suggested that the collective cellular dynamics of plant mitochondria can resolve a tension between mitochondrial proximity and spacing (Chustecki *et al.*, 2021). Mitochondria need to be physically proximal to allow membrane fusion and mixing of contents including mtDNA (Arimura *et al.*, 2004; Sheahan *et al.*, 2005; Rose, 2021). In addition to this exchange, mitochondrial proximity facilitates metabolic exchange and mitochondrial quality control, a process reliant on cycles of fission and fusion, key for maintaining a healthy chondriome (Jones, 1986; Karbowski and Youle, 2003; Arimura *et al.*, 2004; Logan, 2006a; Takanashi *et al.*, 2006; Twig *et al.*, 2008; Liu *et al.*, 2009; Figge *et al.*, 2012; Shutt and McBride, 2013; Agrawal *et al.*, 2018). There are also many other functional implications of inter-mitochondrial proximity including an influence on membrane potential (Santo-Domingo *et al.*, 2013), cristae alignment (Picard *et al.*, 2015), and calcium waves (Ichas *et al.*, 1997). However, there are also benefits to mitochondria remaining physically spaced, including for energy demand, inter-organellar co-localization, and the regulation of metabolic demands (Chen and Chan, 2006; Seguí-Simarro and Staehelin, 2009; Bauwe *et al.*, 2010; Sage *et al.*, 2012; Liesa and Shirihai, 2013; Spillane *et al.*, 2013; Shai *et al.*, 2016; Yu *et al.*, 2016; Schuler *et al.*, 2017; Yu and Pekkurnaz, 2018). The mitochondrial population thus faces a tension between maintaining even spacing of mitochondria and supporting inter-mitochondrial encounters.

Chustecki *et al.* (2021) explored this trade-off between even spacing and supporting encounters by characterizing the ‘social networks’ of the dynamic cellular population, allowing the analysis of connectivity across the chondriome—the whole population of mitochondria in a cell (Logan, 2010). Physical and network analysis revealed that wild-type Arabidopsis uses mitochondrial dynamics to resolve this tension, with mitochondrial motion allowing transient encounters between organelles—and facilitating efficient exchange through the population—while also retaining physical spacing. The development of this approach allows targeted, quantitative questions to be asked about how collective mitochondrial behaviour responds to different situations. In particular, the question of whether and how the cell may control this behaviour in the face of genetic challenges to the mtDNA population remains open (Johnston, 2019). That is, if mtDNA integrity is compromised, can the cell compensate—at least in part—through adapting its control of mitochondrial dynamics?

Here, we pursue this question by investigating the collective behaviour of mitochondria in the *msh1* mutant. Here, MutS HOMOLOGUE 1 (MSH1), responsible for recombination surveillance and repair of organellar genomes (Martínez-Zapater *et al.*, 1992; Abdelnoor *et al*., 2003, 2006; Shedge *et al.*, 2007; Arrieta-Montiel *et al.*, 2009; Davila *et al.*, 2011; Wu *et al.*, 2020) and the rapid segregation of mtDNA heteroplasmy (Broz *et al.*, 2022), is compromised. Disruption of mitochondrial-localized MSH1 leads to an increase in single nucleotide variants and insertion–deletion mutations in mtDNA (Wu *et al.*, 2020), and MSH1 disruption can also lead to substoichiometric shifting in the mitochondrial genome (Martínez-Zapater *et al.*, 1992; Sakamoto *et al.*, 1996; Abdelnoor *et al.*, 2003) [although the full molecular mechanism of MSH1 action on the mitochondrial genome is still not fully characterized (Fukui *et al.*, 2018; Wu *et al.*, 2020), multiple studies support the model of MSH1 influencing double-strand break repair (Davila *et al.*, 2011; Christensen, 2014; Wu *et al.*, 2020)]. *msh1* does not exclusively affect mtDNA: chloroplast maintenance is also compromised (Wu *et al.*, 2020), and downstream metabolic influences of the resulting organelle dysfunction also contribute to the phenotype (Xu *et al.*, 2011, 2012; Virdi *et al.*, 2015; Shao *et al.*, 2017).

Disruption of MSH1 thus provides genetic challenges to the mtDNA and plastid DNA (ptDNA) populations (as well as resultant metabolic and other stresses). We set out to investigate whether these challenges had the effect of changing the collective cellular behaviour of mitochondria. Following the above, we hypothesized that the plant cell might respond physically to compromised mtDNA maintenance, specifically by sacrificing spacing to facilitate more encounters and thus more exchange of mtDNA, and other mitochondrial contents, to compensate for the loss of genetic integrity and accompanying metabolic challenges. As described below, we explored this question by using single-cell microscopy, computational analysis, and network science approaches to characterize and analyse mitochondrial behaviour in *msh1* compared with wild-type Arabidopsis and other mutants.

## Materials and methods

### Plant lines

An MSH1 (previously CHM1-1) ethyl methanesulfonate-derived mutant line in the Columbia background generated by G. Redei (Rédei, 1973) was obtained from the Arabidopsis stock centre (N3372, http://arabidopsis.info/StockInfo?NASC_id=3372). This line carries a single nucleotide polymorphism (SNP) in the fourth exon of genomic region AT3G24320, leading to a non-synonymous glutamate→stop codon change. This line was originally isolated in a *gl1* marked plant, a linkage gene in the third chromosome, and so carries a *gl1* polymorphism, and lacks trichomes. There is evidence to suggest that *gl1* does not alter mitochondrial behaviour (Islam *et al.*, 2020), and the gene is highly expressed in only the early shoot apical meristem (SAM), young leaf, and young flower, not in the hypocotyl used in this study (Nakabayashi *et al.*, 2005; Schmid *et al.*, 2005; Klepikova *et al.*, 2016). This mutant has been used in previous studies as a disruptor of normal MSH1 function (Xu *et al.*, 2011; Wu *et al.*, 2020). Seeds of *Arabidopsis thaliana* with mitochondrial matrix-targeted green fluorescent protein (GFP), and the mtGFP-*friendly* (Mito-GFP::*fmt*) line were kindly provided by Professor David Logan (Logan and Leaver, 2000; El Zawily *et al.*, 2014).

### Crossing and DNA extraction

*msh1* and mtGFP seeds were surface sterilized in 50% (v/v) household bleach solution for 4 min with continual inversion, rinsed three times with sterile water, and plated onto half-strength Murashige and Skoog (1/2 MS) agar. Plated seeds were stratified in the dark for 2 d at 4 °C. Seedlings were grown in 16 h light/8 h dark at 21 °C for 4–5 d, before being transferred to 4:2:1 compost–vermiculite–perlite mixture, and grown until the first flower buds developed. For day/night experiments, seedlings were grown at 22 °C in growth cabinets at 16 h light/8 h dark set to be mid-way (8 h) through the light period or mid-way through the dark period (4 h) at the point of imaging.

The crossing technique followed the protocol of Browse *et al.* (1993), with mtGFP plants as the pollen donor and *msh1* plants accepting. Pollinated stigmas were wrapped gently in plastic wrap, and siliques were left to develop. F_2_ seeds were sown onto 50 μg ml^–1^ kanamycin 1/2 MS (Murashige and Skoog) plates, selecting for individuals carrying the fluorescence construct (Logan and Leaver, 2000), and grown on soil as before. Leaf samples were taken for DNA extraction from all except F_2_ seeds.

Quick DNA extraction was performed on young leaf samples (2–3 weeks old, age dependent on growth rate). Leaf samples were macerated with a pipette tip in 40 µl Extraction Buffer (2.5 ml of 2 M Tris–HCl, 500 µl of 1 M EDTA, 6.25 ml of 2 M KCl, made up to 50 ml with BPC water). The sample was then incubated in a heat block for 10 min at 95 °C. A 40 µl aliquot of dilution buffer was added [3% BSA (1.5 g in 50 ml), filter sterilized], and samples were spun down at 13 000 rpm for 60 s before storing at –20 °C.

### Genotyping and sequencing

For genotyping, primer set 1 was used. A reverse primer (RP1, 5ʹAAACTTCGCGTGGAAACCTTGACTTAATGT 3ʹ) running into the SNP site was designed using dCAPS finder 2.0 (Neff *et al.*, 1998), and the forward primer (FP1, 5ʹCATCTCACCTTCTAGATGTCAGCCTTT 3ʹ) was designed 200 bp upstream of the restriction site. By design, *Bsr*GI will cut a region of 30 bp from the 293 bp element if the SNP is present, producing one larger (260 bp) and one smaller (~30 bp) fragment compared with the wild-type single fragment (293 bp). After PCR amplification, half (5 µl) of the PCR product for each sample was directly added to 1.5 µl of Cutsmart buffer (NEB), 0.2 µl of *Bsr*GI restriction enzyme (NEB), and 8.3 µl of nuclease-free H_2_O. Samples were then incubated at 37 °C overnight, before alternate undigested and digested samples were loaded for gel electrophoresis.

To sequence *MSH1*, the region of interest was first amplified by PCR using primer set 2 (FP2: 5ʹTTGGACCCTAGCTTGAGGAA3ʹ, RP2: 5ʹATCGAAGACCACCAAAAGGA3ʹ) and Phusion high-fidelity DNA polymerase (NEB CAT#M0530S). PCR products were then purified using the QIAquick PCR Purification Kit (Qiagen) and sequenced from primer FP2 using an ABI 3730 capillary sequencer (Applied Biosystems).

### Imaging and video analysis

Seedlings for imaging were sterilized, stratified, and grown on 50 μg ml^–1^ kanamycin 1/2 MS plates as described above. After 4–5 d, seedlings were taken for imaging and, prior to mounting, stained with 10 µM propidium iodide (PI) solution for 3 min to capture the cell wall. Simple mounting of whole seedlings on microscope slides with coverslips was used (modified from Whelan and Murcha, 2015). In order to minimize the effects of hypoxia and physical stress on the seedling, imaging was undertaken in <10 min after the coverslip was added. For day/night experiments, care was taken to expose ‘night’ samples to as little light as possible during imaging sample preparation.

We used a Zeiss 710 laser scanning confocal microscope for imaging of seedlings. To characterize cells, we used an excitation wavelength of 543 nm, detection range 578–718 nm for both chlorophyll autofluorescence (peak emission 679.5 nm) and PI (peak emission 648 nm). For mitochondrial capture, we used an excitation wavelength of 488 nm, detection range 494–578 nm for GFP (peak emission 535.5 nm). Time-lapse images were taken, and all samples used in this study have the same time interval between frames, and the same length of capture, allowing for direct comparison.

For image analysis, single cells were cropped using the PI cell wall outline with Fiji (Image J 2.0.0). The universal length scale of 5 pixels µm^–1^ was applied across all samples. To counter the occasional sample drift within time-lapse videos, drift correction was applied with default settings, using the cell outline via the PI channel as the stability landmark (Correct 3D drift, FIJI, ImageJ 2.1.0; Parslow *et al.*, 2014).

Following Chustecki *et al.* (2021), tracking of individual mitochondria was done using Trackmate (Tinevez *et al.*, 2017) in ImageJ 2.0.0. The LoG detector was used, with typical settings being 1 µm blob diameters (the typical size of a mitochondrion), although 0.8 µm was occasionally used for lower signal samples. The detection threshold was set between 1.5 and 8, and filters were applied on spots if necessary. The Simple LAP Tracker was run with a linking max distance of 4 μm (3 µm used for a few samples), gap-closing distance of 5 μm (4 µm used for a few samples), and gap-closing max frame gap of two frames. For each sample, the quality of overlaying detection for mitochondria was scrutinized, and occasional tracks were edited for precision.

### Physical statistics

Speed (μm per frame) was computed as the distance moved per frame per trajectory. This value is averaged over all trajectories from the duration of the video. Inter-mitochondrial distance is the minimum Euclidean distance (µm) between every mitochondrion and its nearest physical neighbour in each frame. This value is averaged over all frames of the video. Co-localization time is the number of frames any two mitochondria have spent within a threshold distance (1.6 µm) of each other, averaged over all frames.

Mitochondrial morphology analysis was done with Fiji (Image J 2.0.0). Assessing mitochondrial size with fluorescence microscopy may be complicated by overexposure or other differences in signal intensity between samples. To introduce a technical control for exposure of GFP signal between the two genotypes, mtGFP samples were imaged at various gain values for the GFP channel. The images taken at exposures most comparable with the mtGFP-*msh1* images were then identified. This was achieved by comparing intensity distributions across mitochondrial regions and selecting the mtGFP set with the intensity distribution most comparable with mtGFP-*msh1*. In all cases, the mean intensity of mtGFP images selected in this way was within 5% of the mtGFP-*msh1* mean value. Area values (µm^2^) were then taken for mtGFP-*msh1* and selected mtGFP samples, by drawing selection regions around individual mitochondria that were not part of a cluster or directly adjacent to another individual.

Chloroplast co-localization analysis began with parallel tracking of the movement of mitochondria and chloroplasts over time in each sample (Trackmate; Tinevez *et al.*, 2017). Typical settings were the LoG detector using a blob diameter of 1–3 µm, with a detection threshold of 0.8–4, and filters applied on spots if necessary. Linking was done with the Simple LAP tracker with a linking distance of 3 µm or 4 µm, a gap closing distance of 3 µm or 4 µm, and a gap-closing max frame gap of two frames. Tracks were occasionally edited for precision before exporting.

We defined a statistic reporting the propensity of mitochondria to co-localize with chloroplasts beyond the co-localization that would be expected through a random arrangement of organelles. Co-localization ‘enrichment’ *E* is defined here as the ratio of mitochondrial density in chloroplast-adjacent regions to the density in non-adjacent regions, calculated per video frame as


$$\begin{array}{l} E=\left(N_{c}/A_{c}\right)/\left[\left(N-N_{c}\right)/\left(A-A_{c}\right)\right] \end{array}$$

Where *N* is the number of mitochondria in the current frame, *A* is the cell area estimate (µm^2^), *N*_c_ is the number of mitochondria within distance *d* of the centre of the nearest chloroplast, where *d* is 2 × 1.5 µm (twice the typical chloroplast radius). *A*_c_ is the estimate of available area of cell within distance *d* of the centre of a chloroplast (π*d*^2^). *E* therefore reports the relative chloroplast-adjacent mitochondrial density with respect to chloroplast-distant density. Positional data were taken from mitochondrial and chloroplast trajectories as output from Trackmate.

### Network statistics

Encounter networks are built from the close associations of mitochondria. A threshold distance of 1.6 µm was used to define a characteristic close association, being just over one mitochondrion’s length. Lower threshold distances can also be used, yielding fewer encounters, but similar connectivity trends (Chustecki *et al.*, 2021). Networks build up as encounters (edges) between mitochondria (nodes) and are registered over time.

The mean degree is the number of immediate neighbours each node has, averaged over the number of nodes in the network. Network efficiency is the average, over all pairs of nodes, of the reciprocal shortest distance between each pair:


$$\begin{array}{l} E\left(G\right)=\frac{1}{n\left(n-1\right)}\sum_{i\neq j\in G}\frac{1}{d\left(i,j\right)} \end{array}$$

where *G* is the network of interest, *n* is the number of nodes in the network, and *d*(*i, j*) is the distance (edge number) between node *i* and node *j*. The graph diameter is the length of the longest direct path across the network, a quantification of the number of edges connecting the two furthest nodes across a network. The mean graph betweenness centrality is the average number of shortest paths crossing each node in the network. The mean connected component number is the average number of disconnected subgraphs within the network.

## Results

### Construction, genotyping, and phenotyping of mtGFP-*msh1*

To allow the visualization of mitochondrial dynamics in the *msh1* mutant, we created mtGFP-*msh1*, combining the transgenic mtGFP line where GFP is localized to mitochondria [from an original line kindly provided by Professor David Logan (Logan and Leaver, 2000)] with a mutant line where MSH1, an organelle genome maintenance factor, is perturbed by a premature stop codon caused by an SNP (Abdelnoor *et al.*, 2003; see the Materials and methods for more details). We verified the crossed line using derived cleaved amplified polymorphic sequence (dCAPS) genotyping for the SNP and rosette phenotyping for characteristic variegation in the *msh1* line (Supplementary Fig. S1), where in contrast to both wild-type mtGFP and Col-0, mtGFP-*msh1* retained the expected variegated and low growth phenotype of the *msh1* mutant (Supplementary Fig. 2A–C). The candidate line at F_3_ showed the presence of the SNP (Supplementary Fig. S1A), as well as resistance to kanamycin, demonstrating the presence of the mtGFP transgene (Logan and Leaver, 2000). Sequencing of the F_3_ candidate line confirmed the presence of the SNP in the region encoding *MSH1* (Supplementary Fig. S3). Sequencing of three F_4_ candidate line offspring also showed the presence of the SNP, validating the genetic makeup of the mtGFP-*msh1* mutant.

### *msh1* alters physical dynamics of mitochondria

**Fig. 1.**
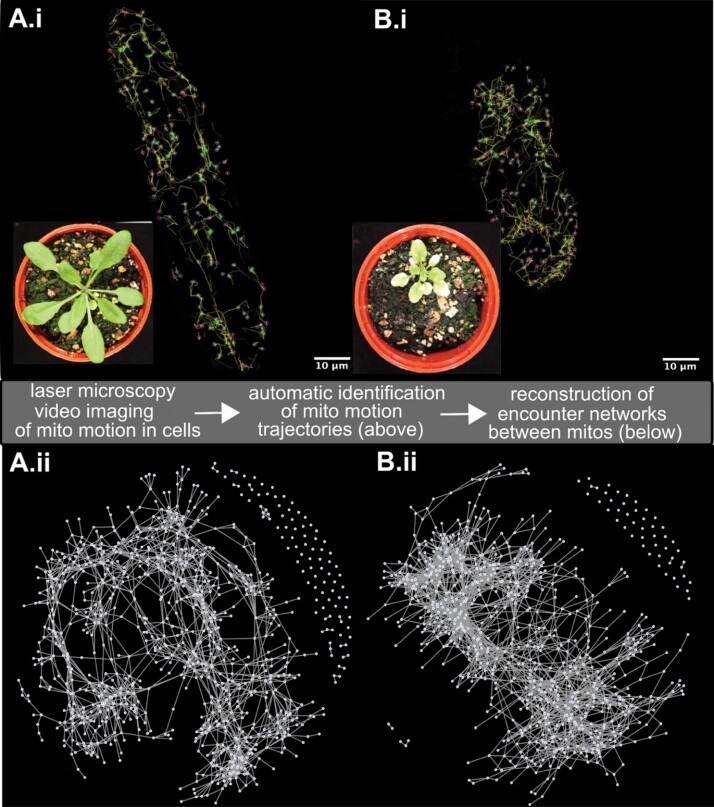
Characterizing the ‘social networks’ of plant mitochondria in mtGFP (A) and mtGFP-*msh1* (B). Top panels (i) illustrate the tracking process of (green) fluorescent mitochondria in single hypocotyl cells from seedlings, using Trackmate (Tinevez *et al.*, 2017). Mitochondria are automatically identified (pink spots, diameter 1 µm), and computed tracks over time are shown [for clarity, only 10 local frames are shown (yellow)]. Insets show whole-plant phenotypes of the two lines later in development. Bottom panels (ii) show the networks of mitochondrial encounters corresponding to the single-cell dynamics (nodes are mitochondria, edges are encounters), built up over a time window of observation (here 233 s).

Following the creation of mtGFP-*msh1*, we used confocal microscopy to characterize mitochondrial dynamics in single hypocotyl cells of 4- to 5-day-old seedlings in this mutant, and compared these dynamics with those of the mtGFP transgenic line, representing wild-type mitochondrial motion. This imaging approach followed the protocol from Chustecki *et al.* (2021). Briefly, we recorded time-lapse videos of mitochondrial motion in single cells, and computationally identified trajectories of individual mitochondria using TrackMate (Tinevez *et al.*, 2017). From these trajectories, we can analyse individual and collective behaviour of mitochondria, including speeds, co-localizations, and many more statistics (Chustecki *et al.*, 2021). Figure 1 illustrates the process of tracking fluorescent mitochondria over time, in representative mtGFP (Fig. 1Ai) and mtGFP-*msh1* (Fig. 1Bi) single cells. Generally and qualitatively, as with wild-type mtGFP mitochondrial motion, mtGFP-*msh1* mitochondria showed a mixture of diffusive and ballistic motion, with some organelles remaining static, and others moving swiftly across the cell. These organelles also co-localize with one another, and occasionally co-localize with chloroplasts (Supplementary Video S1).

**Fig. 2.**
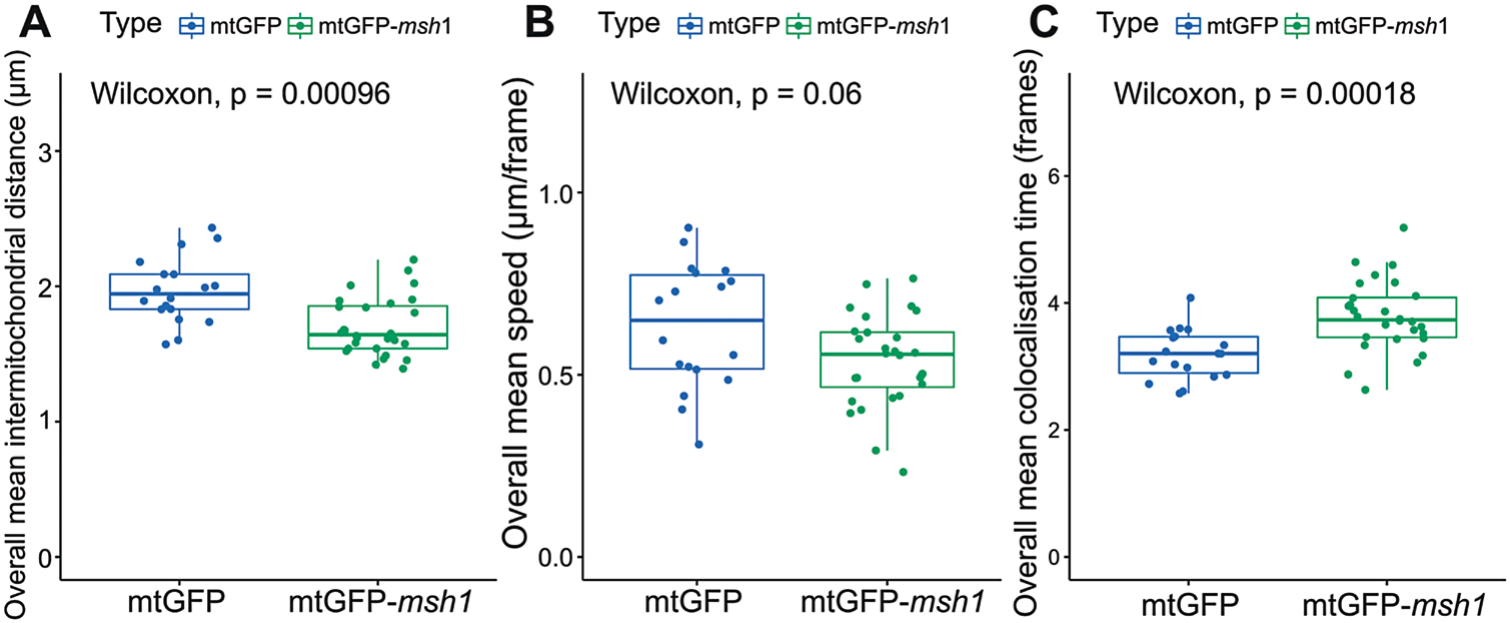
Physical summary statistics differ between mtGFP and mtGFP-*msh1*. Each point represents a summary statistic for one cell (mtGFP *n*=18, mtGFP-*msh1 n*=28). *P*-values represents outcome of the Wilcoxon rank sum test across both genotypes, without multiple hypothesis correction. Boxplots represent the median and 25th/75th percentile, with whiskers showing the smallest/largest value within 1.5× the interquartile range. Each individual point gives the mean statistic across an entire video, corresponding to 233 s of video time.

We found that mitochondria in mtGFP-*msh1* on average were less evenly spread and were physically associated for longer times in hypocotyl cells (Fig. 2). Mean inter-mitochondrial distance, reporting the average distance (in microns) to the nearest physical neighbour in the cell, was lower in mtGFP-*msh1*, reflecting a less evenly spread population (Fig. 2A). The median speed of individual mitochondria in mtGFP-*msh1* was also lower, although differences between the lines did not cross a significance threshold when we used a conservative non-parametric test (Fig. 2B). Co-localization time, reporting the time over which two mitochondria are within a threshold of each other, was higher in mtGFP-*msh1* (Fig. 2C). Other physical and temporal aspects of mitochondrial behaviour were not dramatically different in the *msh1* mutant. Cell sizes were similar across all lines (Supplementary Fig. S4), suggesting that these physical differences are intrinsic properties of the mitochondrial population and not a result of altered cellular morphology. Mitochondrial dynamics did not differ substantially when observed in night and day cycles either within or between either genotype (Supplementary Fig. S5A–G). We did observe a small change in individual mitochondrial area: *msh1* mitochondria were slightly smaller (Supplementary Fig. S5H, I). An increase in mitochondrial size in white (variegated) tissue in the *msh1* mutant has been previously observed (Xu *et al.*, 2011), reflecting a different direction of effect in tissue with a presumably different metabolic poise from the hypocotyl cells we consider. The propensity of mitochondria to co-localize with chloroplasts—measured as the relative density of mitochondria in chloroplast-adjacent to chloroplast-distant regions (see the Materials and methods)—did not significantly change between genotypes (Supplementary Fig. S5J). As always, absence of evidence for effects here cannot be interpreted as evidence of absence of an effect, and these features may in fact differ between genotypes—but the scale of these differences was not large enough to be detectable here, suggesting that the collective physical dynamics we observe are the larger magnitude effect. The changes in collective behaviour that we do observe are thus compatible with our hypothesis that the cell sacrifices physical spacing (to favour organelle encounters allowing exchange of contents) in the *msh1* mutant.

### Alterations in physical dynamics of *msh1* affect social dynamics

To explore whether this change in spacing could indeed facilitate inter-mitochondrial connectivity, we next characterized the ‘encounter networks’ of mitochondria, defined as the set of co-localizations between pairs of mitochondria that occur within a given time frame (see the Materials and methods, Fig. 1Aii, Bii; Supplementary Fig. S6). Akin to social networks, describing social interactions between individuals in a population, these encounter networks shape the potential for beneficial exchange of contents across the mitochondrial population (Chustecki *et al.*, 2021).

Salient features of these encounter networks for potential exchange of mitochondrial contents are the degree distribution (the number of different mitochondria each mitochondrion encounters), the diameter of the network (the length in edges of the longest direct route across the network), and the network efficiency. This final quantity is the average of the reciprocal lengths of the shortest paths between each pair of mitochondria in the network. If all pairs of mitochondria are connected by short paths (facilitating exchange through the network), reciprocal lengths, and network efficiency, are high. If some pairs are connected only by long paths, or are disconnected, reciprocal lengths and efficiency are low and information exchange is more challenging.

**Fig. 3.**
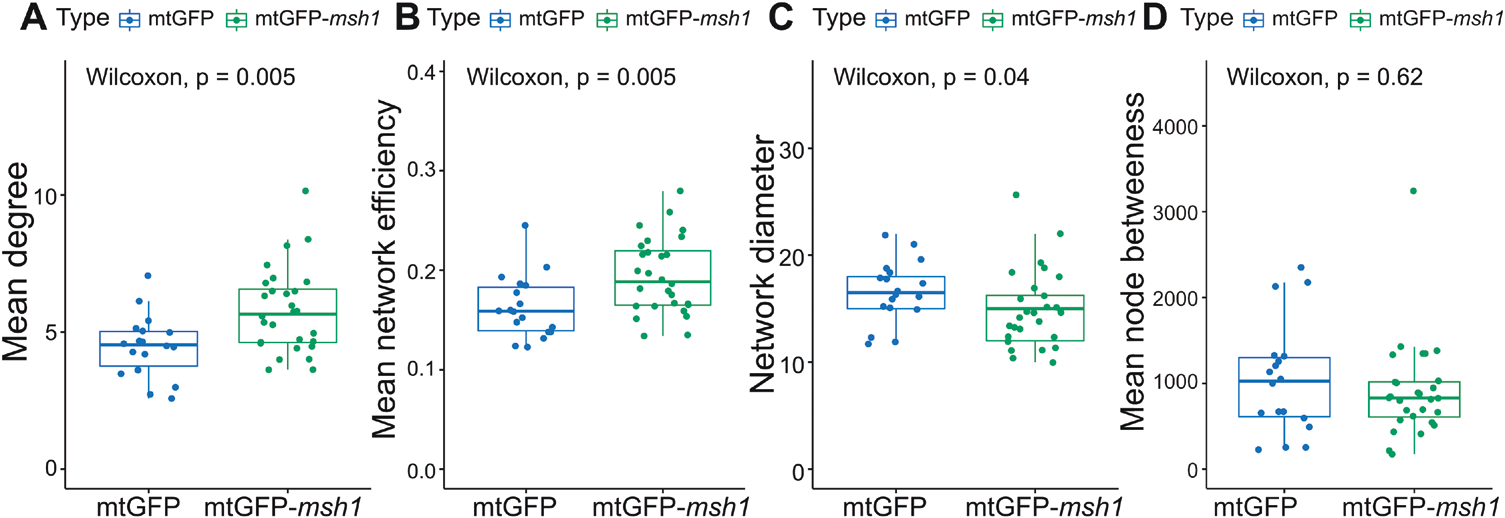
Social summary statistics differ between mtGFP and mtGFP-*msh1.* Each point represents a summary statistic for one cell (mtGFP *n*=18, mtGFP-*msh1 n*=28). *P*-values represents outcome of the Wilcoxon rank sum test across both genotypes, without multiple hypothesis correction. Boxplots represent the median and 25th/75th percentile, with whiskers showing the smallest/largest value within 1.5× the interquartile range. Each individual point is from a network corresponding to an observed time window of 233 s.

We found that the encounter networks of mtGFP-*msh1* had a higher mean degree and higher efficiency than the mtGFP line (representative of wild-type mitochondrial networks) (Fig. 3A, B). Mitochondria in the *msh1* mutant are thus more directly connected through encounters, facilitating easier exchange of contents. Network diameter is also shorter across mtGFP-*msh1* networks, again suggesting increased organelle connectivity; but we note the significant difference was not retained after multiple hypothesis testing (Fig. 3C). The size of networks, quantified either by node or edge number, remained similar between mtGFP and mtGFP-*msh1* over time (Supplementary Fig. S7). There was no significant difference across values for betweenness centrality, an average of the number of shortest paths crossing each node in the network (Fig. 3D).

These network statistics are time dependent, because networks build up over time as more encounters between individuals occur. As seen in Supplementary Fig. S8, *msh1* differences in degree value remain across different observation time windows, with network efficiency differences significant at later frames (Fig. 3A, B; Supplementary Fig. S8A, B), when networks have built up with more encounters. Network diameter relationships across the lines do not substantially change over time, but betweenness centrality is significantly different for early comparisons between lines, although not at later frames (Fig. 3C, D; Supplementary Fig. S8C, D). This could be a consequence of the topology of smaller networks, before so many encounters and connections between smaller cliques of mitochondria are formed.

Taken together with the physical results, these observations support our hypothesis that the genetic challenge provided by the *msh1* mutation can invoke a compensatory shift in mitochondrial dynamics, sacrificing physical spacing to facilitate more organelle encounters, which may in turn support more efficient exchange of contents.

### The collective dynamic response to *msh1* resembles the response to *friendly*

We next asked whether the altered mitochondrial behaviour in the face of the *msh1* perturbation shared similarities with altered behaviour under a physical perturbation to mitochondrial dynamics. To this end, we characterized an mtGFP-*friendly* mutant within which the fusion of these organelles is perturbed (El Zawily *et al.*, 2014), increasing the association time between individuals, and posing a transient challenge to the social connectivity and physical spread trade-off as shown in Chustecki *et al.* (2021). Recent work has illuminated the co-localization of FRIENDLY to depolarized mitochondria as an essential part of the mitophagy pathway (Ma *et al.*, 2021); its perturbation results in reduced mitochondrial fusion, increased mitochondrial clustering, and a wide range of metabolic issues (El Zawily *et al.*, 2014; Ma *et al.*, 2021). This mutant has a pronounced growth phenotype, though more limited than *msh1* (Supplementary Fig. S2D).

**Fig. 4.**
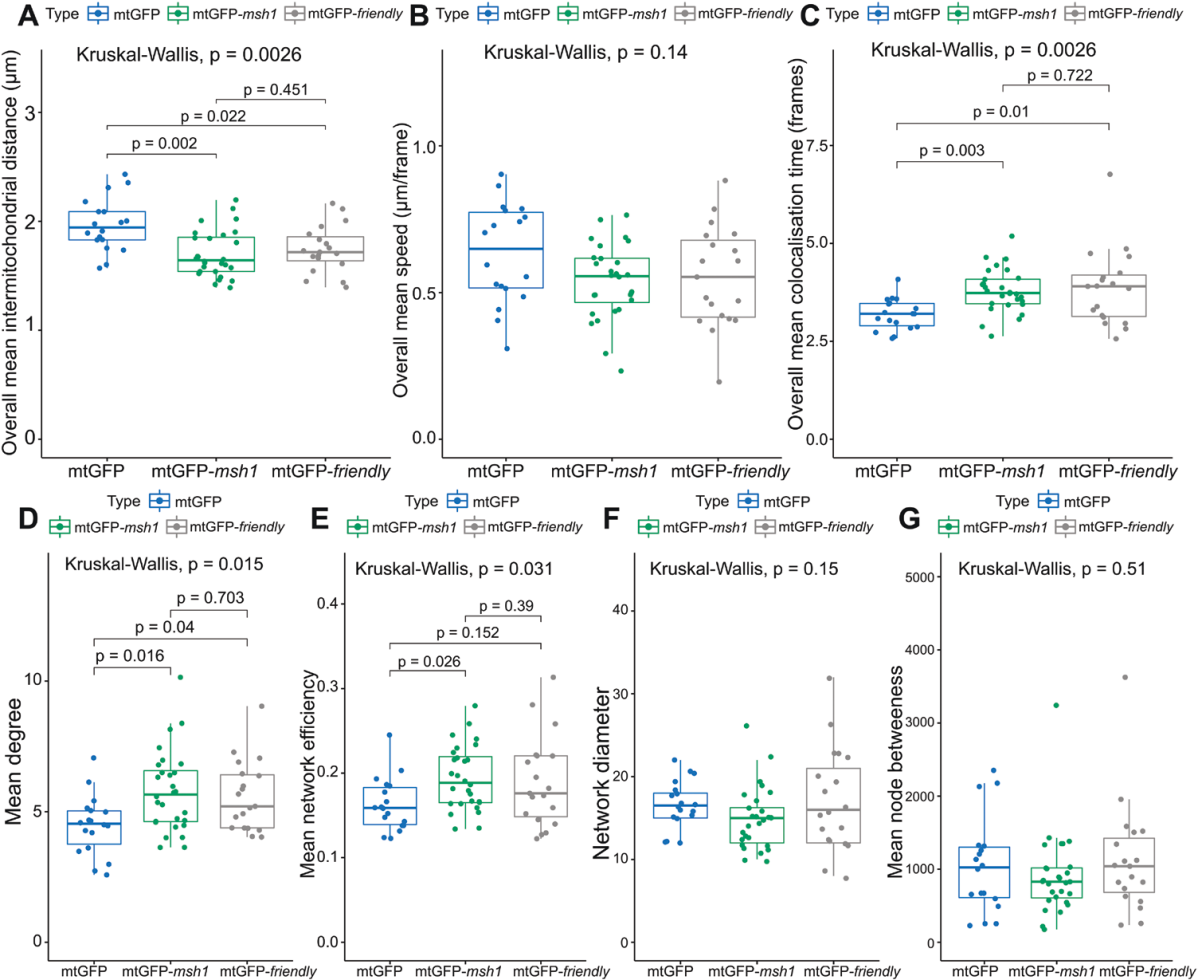
Physical and social summary statistics compared across mtGFP, mtGFP-*msh1*, and *friendly.* Each point represents a summary statistic for one cell (mtGFP *n*=18, mtGFP-*msh1 n*=28, *friendly n*=19). *P*-values represent Kruskal–Wallis test outcomes across all three genotypes, and pairwise *P*-values are false discovery rate-adjusted outcomes of a post-hoc Dunn test, without multiple hypothesis correction across statistics. Boxplots represent the median and 25th/75th percentile, with whiskers showing the smallest/largest value within 1.5× the interquartile range. Each physical datapoint (A–C) is a mean across a 233 s time window, and each social datapoint (D–G) is from a network corresponding to a time window of 233 s.

To explore the relationship between changes in mitochondrial behaviour due to physical and genetic challenges, we compared mitochondrial behaviour in mtGFP, mtGFP-*msh1*, and mtGFP-*friendly*. Strikingly, the physical and social statistics observed in mtGFP-*msh1* and mtGFP-*friendly* lines are remarkably similar, with no statistically detectable differences between these genotypes. Of course, an absence of statistical significance does not imply the absence of an effect, but the observed magnitudes of the statistics and our moderate sample sizes (*n*=28 for mtGFP-*msh1*, *n*=19 for mtGFP-*friendly*) suggest that the behaviours are indeed rather similar (Fig. 4). There was a slightly lower inter-mitochondrial distance alongside an increased degree and network efficiency within mtGFP-*msh1*—suggesting a marginally more pronounced shift towards connectivity—although these observations did not meet a statistical significance threshold for a non-parameteric comparison (Fig. 4A, D, E). Both mutant genotypes show a significantly decreased inter-mitochondrial distance, and increased co-localization time and degree, when compared with wild-type mtGFP (Fig. 4A, C, D).

Previous work (Chustecki *et al.*, 2021) found that the difference between mtGFP-*friendly* and wild-type behaviour diminished over time: initially rather cliquey, the *friendly* networks became more globally connected over time as itinerant mitochondria formed social bridges between cliques. Our statistical analysis here supports this picture for mean degree in both *friendly* and *msh1* (Fig. 4D; Supplementary Fig. S8A) while revealing a more nuanced picture for other network statistics. In particular, network efficiency differences between the mutants and wild type do not diminish over time to the same extent (Fig. 4E; Supplementary Fig. S8B), suggesting that the global changes in collective behaviour are maintained robustly over time despite similarities in local behaviour. Overall, both the magnitudes and time behaviour of collective dynamic changes were quantitatively similar in *friendly* and *msh1*, supporting the comparable influences of the two perturbations.

## Discussion

**Fig. 5.**
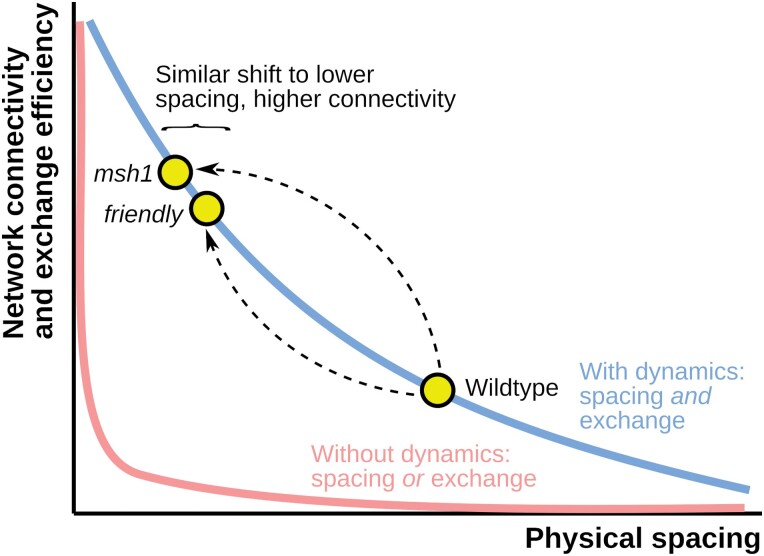
Different resolutions to the social/spacing trade-off. There exists a trade-off (coloured curves) between physical spacing of mitochondria (horizontal axis) and the connectivity of the chondriome (vertical axis). Without mitochondrial dynamics, static organelles are either co-localized or spaced, with little capacity to support both behaviours together (pink). Mitochondrial dynamics provides a resolution: as organelles move, they can transiently co-localize while usually remaining spaced (blue), allowing some capacity for both behaviours. Wild-type Arabidopsis adopts a particular balance between spacing and encounters. This balance is shifted in the *msh1* mutant, where mitochondria sacrifice spacing and increase inter-mitochondrial encounters. This increased ‘social’ connectivity may support fusion, exchange, and complementation of damaged mtDNA. *friendly* mutants reflect a similar shift in balance, caused by a physical perturbation.

Mitochondria across eukaryotes are strikingly dynamic. In some cases, including the delivery of ATP to synapses in neurons (Hollenbeck and Saxton, 2005; Mironov, 2007; MacAskill *et al.*, 2010) and of fit mitochondria to growing buds in yeast (Fehrenbacher *et al.*, 2004; Pernice *et al.*, 2018), the reasons for this motion are largely explained. In many other cases, the advantages and disadvantages of the rich dynamics of mitochondria remain unclear. Here we have demonstrated that a perturbation to nuclear-encoded machinery responsible for mtDNA maintenance influences the collective physical dynamics of plant mitochondria in such a way as to trade reduced spacing for increased connectivity. In turn, we suggest that this increased capacity for interaction may support more mtDNA sharing and complementation in the face of compromised mtDNA (Fig. 5), as well as increased potential exchange of other chemicals. Strikingly, this response of collective dynamics to a genetic challenge resembles that to a physical challenge (induced by the *friendly* mutation), underlining the link between genetic and physical dynamics of mitochondrial populations.

*msh1* mutants demonstrate an increase in single nucleotide variants and insertion–deletion mutations (Wu *et al.*, 2020), as the protein forms part of the mtDNA damage repair machinery (Davila *et al.*, 2011; Christensen, 2014; Gualberto *et al.*, 2014; Wu *et al.*, 2020). In other plants, although not in Arabidopsis, substoichiometric shifting due to MSH1 disruption also leads to cytoplasmic male sterility (Arrieta-Montiel *et al.*, 2001; Sandhu *et al.*, 2007; Xu *et al.*, 2011; Virdi *et al.*, 2016)—a substantial biological challenge (although one of value in crop breeding). The increased connectivity we observe across the chondriome could provide individual mitochondria with a chance to access undamaged mtDNA, or extra copies of gene sequences to use as guide strands during double-strand break repair. One potentially quite general principle is that the physical dynamics of organelles exert control on the genetic dynamics of organellar DNA, and the cell can thus address genetic priorities by controlling physical behaviour (Johnston, 2019). However, other effects of the *msh1* mutation may also play roles in shaping the collective dynamic response, including metabolic influence from mitochondrial and chloroplast dysfunction, transgenerational subtleties of mtDNA mutations and nuclear DNA methylation, and consequent or independent influences on the internal structure of the cell. Further work characterizing mitochondrial collective dynamics in lines controlling for these influences will help provide further support for the physical–genetic feedback hypothesis.

Other examples exist of where plant mitochondrial dynamics may influence mtDNA genetic structure. In the SAM, a cage-like mitochondrial network has been observed to form (Seguí-Simarro and Staehelin, 2009), in contrast to the largely individual mitochondria observed in other tissues. This network structure allows mtDNA mixing and facilitates recombination (Edwards *et al.*, 2021; Rose, 2021). In conjunction with this physical change, relative expression of MSH1 is particularly high in the SAM, which may both assist with maintenance and support germline mtDNA segregation through gene conversion as an evolutionary priority (Schmid *et al.*, 2005; Edwards *et al.*, 2021; Broz *et al*., 2022).

Other tissues where mitochondrial dynamics have been characterized include leaves (where the tight packing of chloroplasts means that mitochondria are extremely constrained), cotyledon [where mitochondrial collective dynamics resemble those observed in hypocotyl (Arimura *et al.*, 2004; Chustecki *et al.*, 2021)], and root epidermis [where some cells appear to have relatively stationary mitochondrial populations, and others have collective dynamics that again resemble those in hypocotyl (Logan and Leaver, 2000; Zheng *et al.*, 2009)—including in response to the *friendly* mutant (El Zawily *et al.*, 2014)]. Speculatively, this suggests a picture where collective dynamics (under the constraints of cell structure) can contribute to mtDNA maintenance in similar ways in somatic tissues, while the above-ground germline reflects the completely connected extreme on the spectrum of connectivity and spacing (Fig. 5) due to the increased need for faithful mtDNA inheritance between generations (Logan, 2006b; Seguí-Simarro and Staehelin, 2009; Woloszynska, 2010; Johnston, 2019; Edwards *et al.*, 2021).

The link between the physical behaviour of mitochondria and the genetic behaviour of mtDNA is still being elucidated (Aryaman *et al.*, 2019; Johnston, 2019; Edwards *et al.*, 2021). The production, degradation, fission, fusion, partitioning, motion, and arrangement of mitochondria in the cell all influence the genetic structure of the mtDNA population. Mitochondria are increasingly being recognized as ‘social’ organelles, with their interactions playing important functional roles beyond what a collection of independent individuals could achieve (Picard and Sandi, 2021). In plants, a picture of collective behaviour emerging from a population of individuals is particularly pertinent, as mitochondria physically retain individual identities to a much greater extent than in other kingdoms where fused networks are common. The sharing of contents between mitochondria, and consequent control of contents throughout the population, is an example of such emergent behaviour that could not be achieved by independent organelles. Our results here demonstrate that the collective dynamics of mitochondria may respond to genetic challenges as well as physical challenges, suggesting that control of these dynamics may provide the cell with a way of exploiting the physical–genetic link in the face of genetic perturbation. Plant cells, with largely individual mitochondria readily visualized in a quasi-2D cytosolic domain, are an excellent model system for further exploring this link, and we believe that the encounter networks we characterize here will find further use in investigating the vital emergent collective dynamics of the chondriome.

## Supplementary data

The following supplementary data are available at *JXB* online.

Fig. S1. Genotyping for F_3_*msh1* homozygosity leads to consistently variegated F_4_ progeny.

Fig. S2. Plant phenotypes reveal developmental differences across genotypes.

Fig. S3. Single nucleotide polymorphism in MSH1 retained in the F_3_ generation of the mtGFP-*msh1* cross.

Fig. S4. No evidence found for a difference between median cell area across genotypes.

Fig. S5. Limited *msh1* influence on other temporal or spatial aspects of mitochondrial behaviour.

Fig. S6. Sample encounter networks for mtGFP and mtGFP–*msh1*.

Fig. S7. Node number and edge number of encounter networks did not vary greatly between lines for mtGFP, mtGFP–*msh1*, and mtGFP*-friendly*.

Fig. S8. Social summary statistics provide evidence of differences between mtGFP, mtGFP–*msh1*, and *friendly*, at three earlier times.

Video S1. Example mitochondrial dynamics in *msh1* hypocotyl.

erac250_suppl_supplementary_Figures

erac250_suppl_supplementary_video_S1

## Data Availability

All data and analysis codes are available from Github at https://github.com/StochasticBiology/plant-mito-dynamics
