# Supplementary material for: Altered collective mitochondrial dynamics in the Arabidopsis msh1 mutant compromising organelle DNA maintenance
Source: J Exp Bot. 2022 Jun 5;73(16):5428–39. doi: 10.1093/jxb/erac250 (PMC9467644; doi:10.1093/jxb/erac250)
Supplement: erac250_suppl_supplementary_Figures [file erac250_suppl_supplementary_figures.pdf]

Altered collective mitochondrial dynamics in the Arabidopsis *msh1* mutant compromising organelle DNA maintenance

Joanna M. Chustecki, Ross D. Etherington, Daniel J. Gibbs, Iain G. Johnston

Supplementary Information

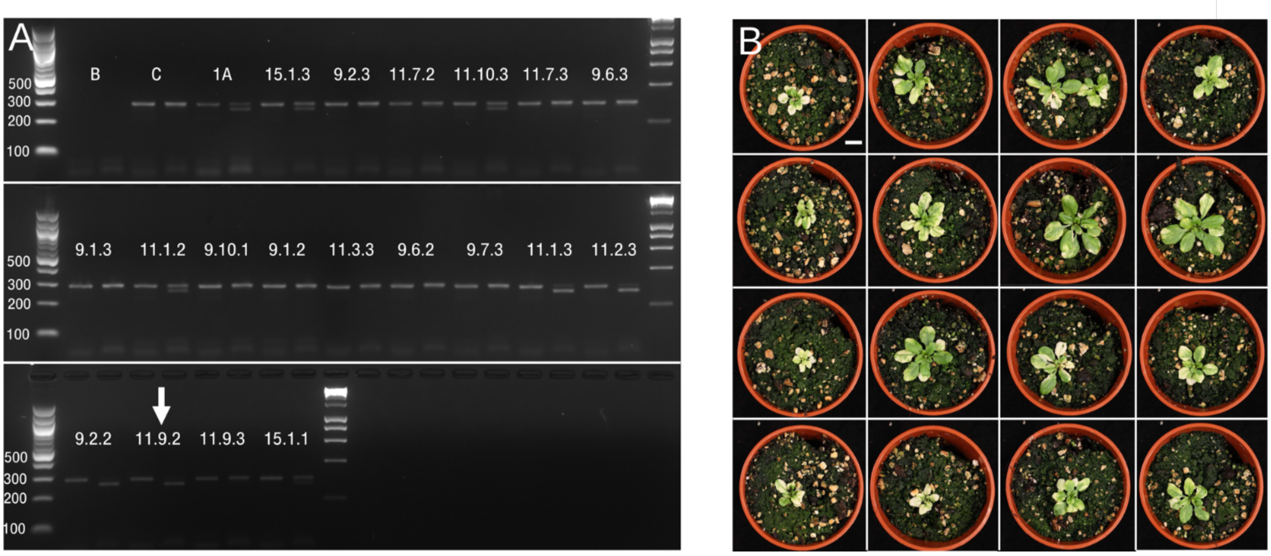

Supplementary Figure 1: Genotyping for F3 *msh1* homozygosity leads to consistently variegated F4 progeny. (A) dCAPS genotyping for WT gives 293bp fragment, but in the presence of *msh1* SNP mutation gives ~260bp fragment, when digested with a restriction enzyme. Each line has an undigested (left band) and a digested (right band) sample. Homozygosity is demonstrated by one upper band (left, 293bp), and one lower band (right, ~260bp). The entire image is one gel, with empty vertical space cropped between sets of lanes. Heterozygosity is demonstrated by one left band and two fragments in the right band. (B) Phenotype of candidate line 11.9.2, showing all individuals with variegated phenotype typical of the *msh1* mutation in *Arabidopsis* (30 days old). Scale bar = 1cm.

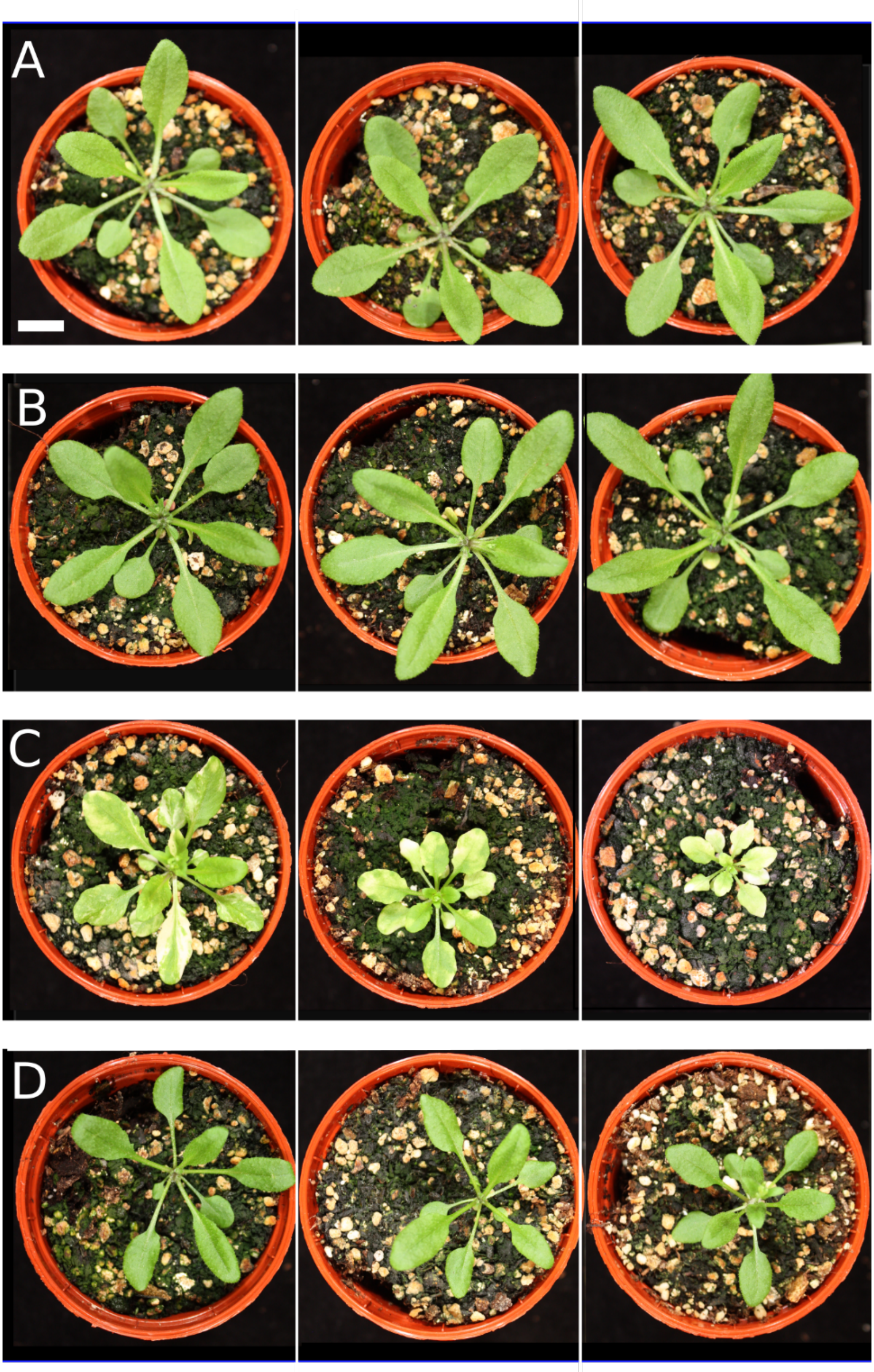

Supplementary Figure 2: Plant phenotypes reveal developmental differences across genotypes. Rosette images of three representative examples of 31 day old plants taken of A) Col-0; B) mtGFP; C) mtGFP-*msh1*; D) mtGFP-*friendly*. Scale bar = 1cm.

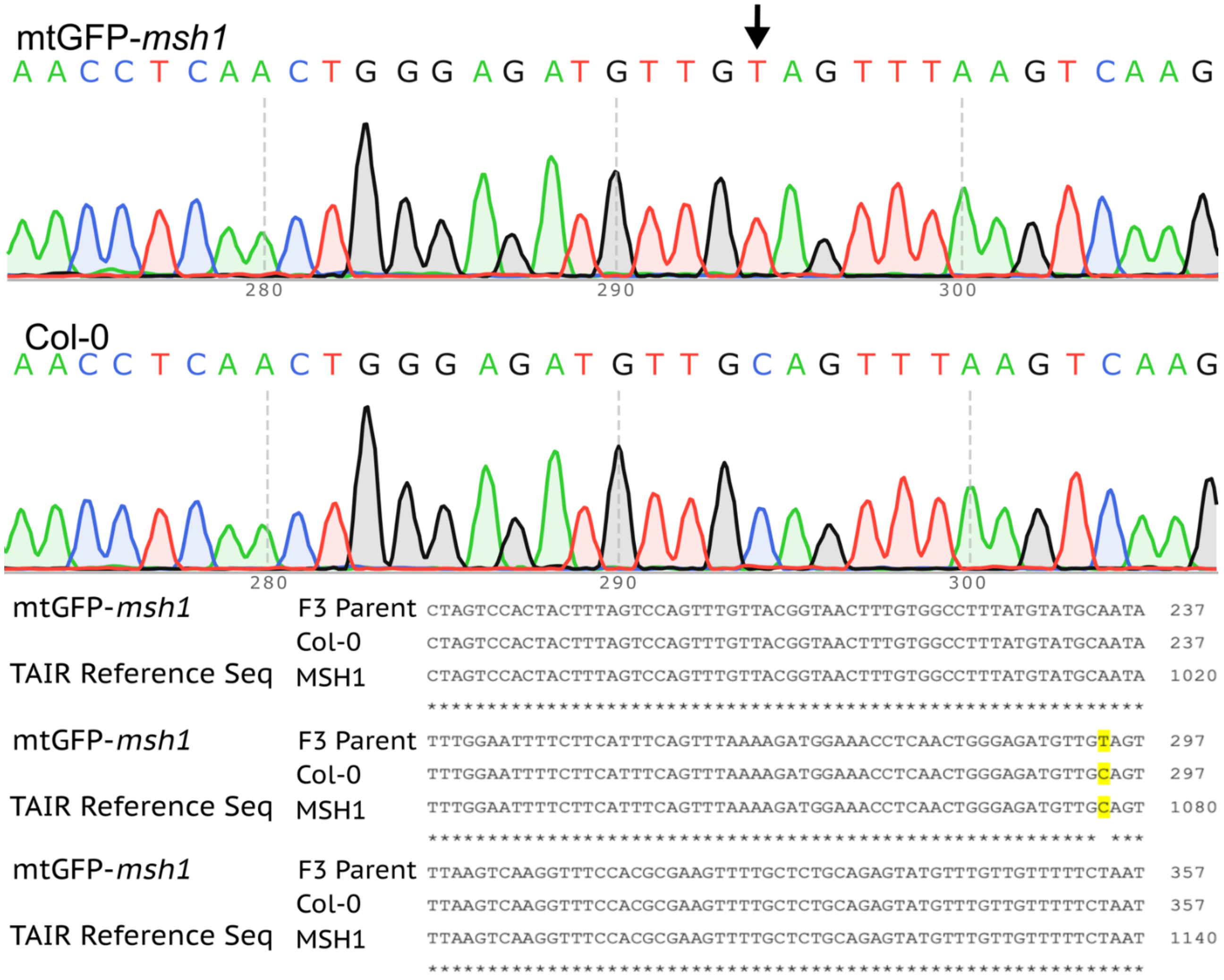

Supplementary Figure 3: Single nucleotide polymorphism in MSH1 retained in the F3 generation of mtGFP-*msh1* cross. Upper panel illustrates single autoscaled peaks, showing base pair reads across the middle of the amplified region and at position 294 (arrow), evidence of a homozygous SNP. Lower panel shows alignment of base pair reads of mtGFP-*msh1* F3 parent, Col-0 sample, and the TAIR reference genome at the *MSH1* gene. Highlighted base shows the SNP leading to CAG (glutamine) to TAG (stop).

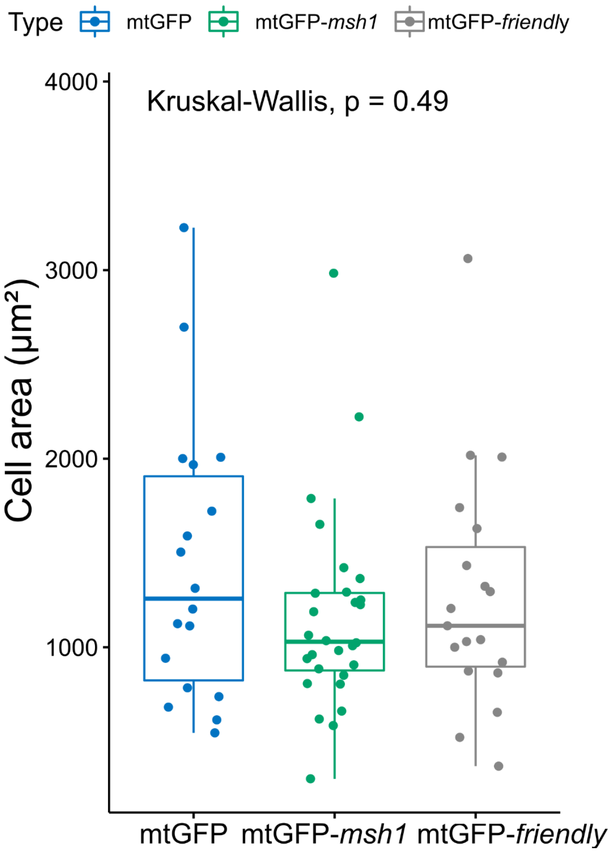

Supplementary Figure 4: No evidence found for a difference between median cell area across genotypes. Comparison of two-dimensional cell area (µm^2) between the three genotypes using the Kruskal-Wallis test. Boxplots represent the median and 25th/75th percentile, with whiskers showing the smallest/largest value within 1.5x the interquartile range. P-value represents Kruskal Wallis test outcome across all three genotypes.

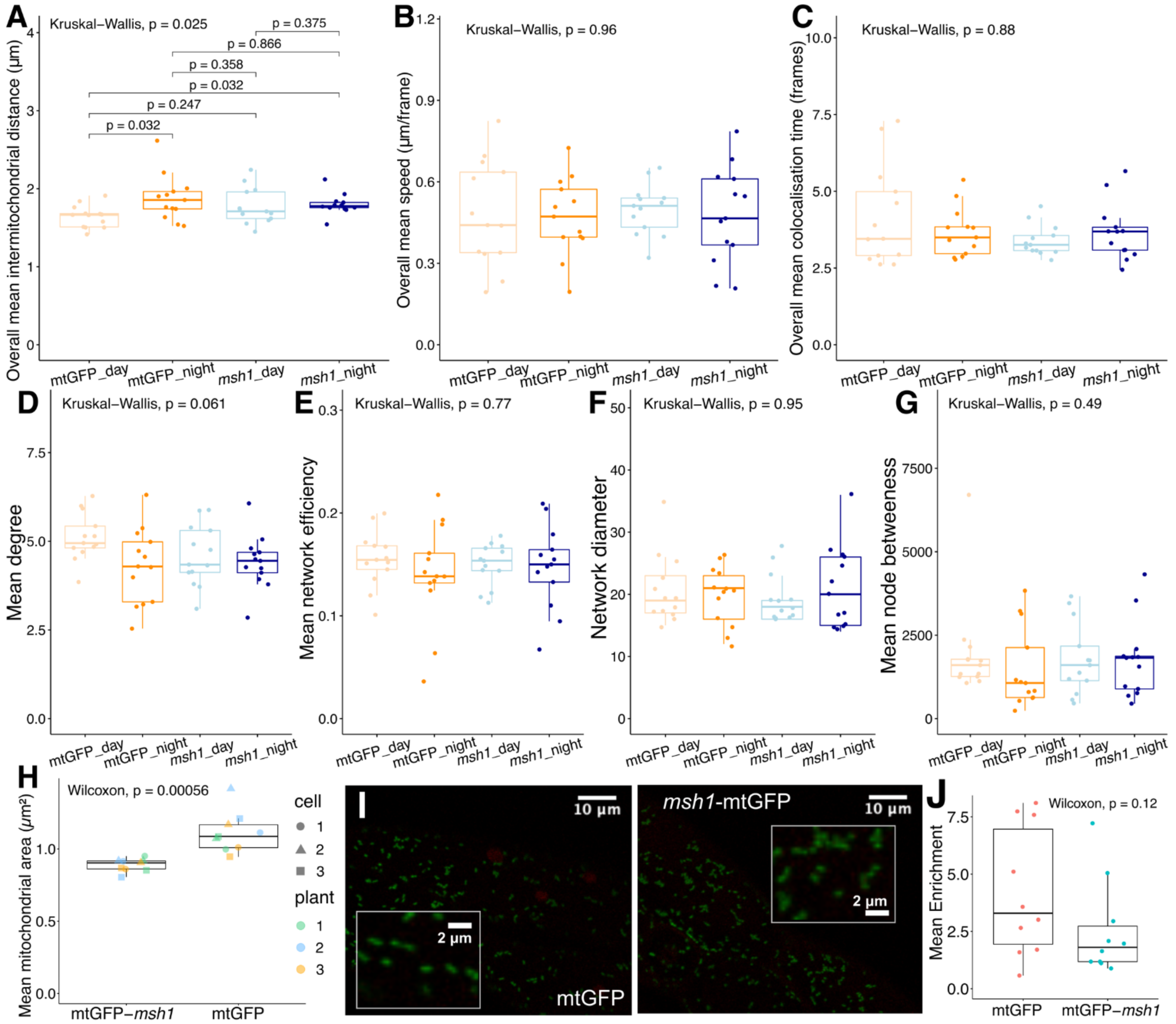

Supplementary Figure 5: Limited *msh1* influence on other temporal or spatial aspects of mitochondrial behaviour. (A-G) Physical and social summary statistics compared between mtGFP and mtGFP-*msh1* imaged during day or night. Each point represents a summary statistic for one cell (*n* = 13). Physical statistics are overall mean values across an entire video, and social statistics are taken from the final network frame built up over video time, both corresponding to 233 seconds. H) Mean area (µm^2) of individual mitochondria compared across genotypes. Each mean value is taken from 25 individual mitochondria in one cell, with three cells (shapes) taken over three seedlings (colours). (I) Snapshots of mtGFP and B) mtGFP-*msh1* hypocotyl cells with mitochondrial matrix localised GFP (green) and Propidium Iodide cell boundary stain (red) also capturing chloroplast autofluorescence (red), illustrating limited size differences. J) Mean colocalization enrichment (*E*) values between mitochondria and chloroplasts (reporting relative chloroplast-adjacent mitochondrial density compared to chloroplast-distant density, see methods) taken for both genotypes (*n* = 10). P-values represents outcome of the Wilcoxon rank sum test across both genotypes. Boxplots represent the median and 25th/75th percentile, with whiskers showing the smallest/largest value within 1.5x the interquartile range.

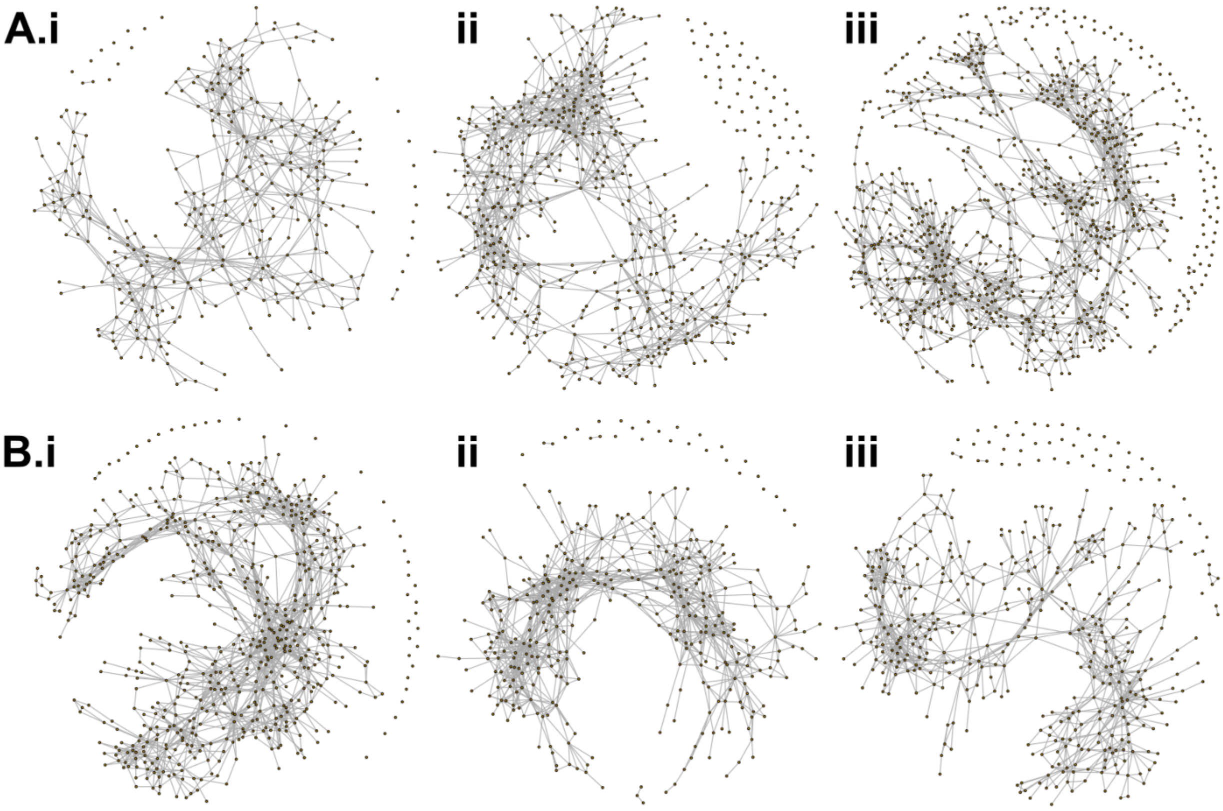

Supplementary Figure 6: Sample encounter networks for mtGFP (A) and mtGFP-*msh1* (B). Networks are built from close encounters (edges) between mitochondria (nodes) (see methods). Networks here are built up from 233 seconds of video time.

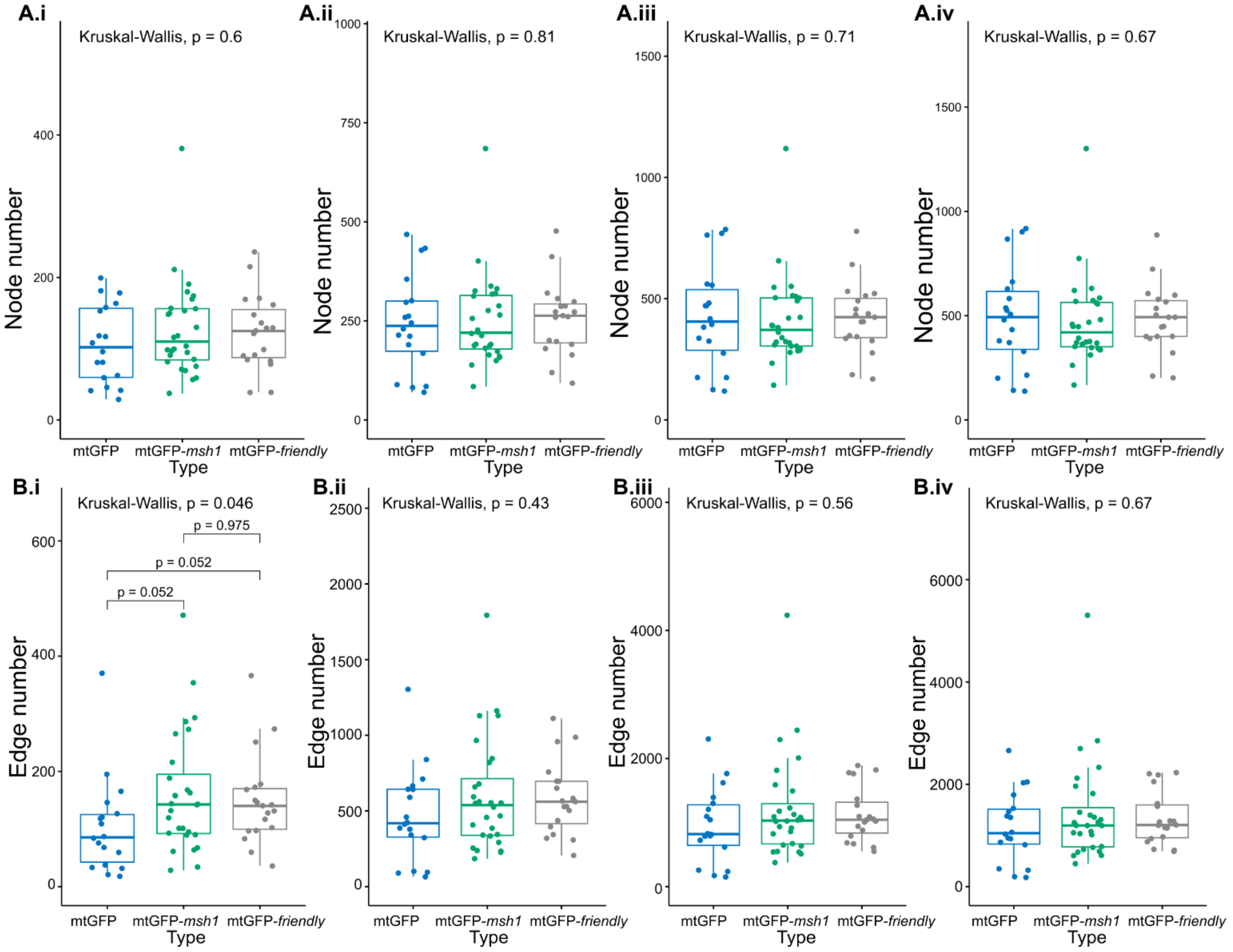

Supplementary Figure 7: Node number (A) and edge number (B) of encounter networks did not vary greatly between lines for mtGFP, mtGFP-*msh1*, and mtGFP-*friendly*. With the exception of edge numbers for early frame 10. From left to right (i-iv) graphs show snapshots of networks at frames 10, 50, 100, 120. P-values represent Kruskal Wallis test outcomes across all three genotypes, and pairwise p-values are false discovery rate adjusted outcomes of a post-hoc Dunn test, without multiple hypothesis correction across statistics.

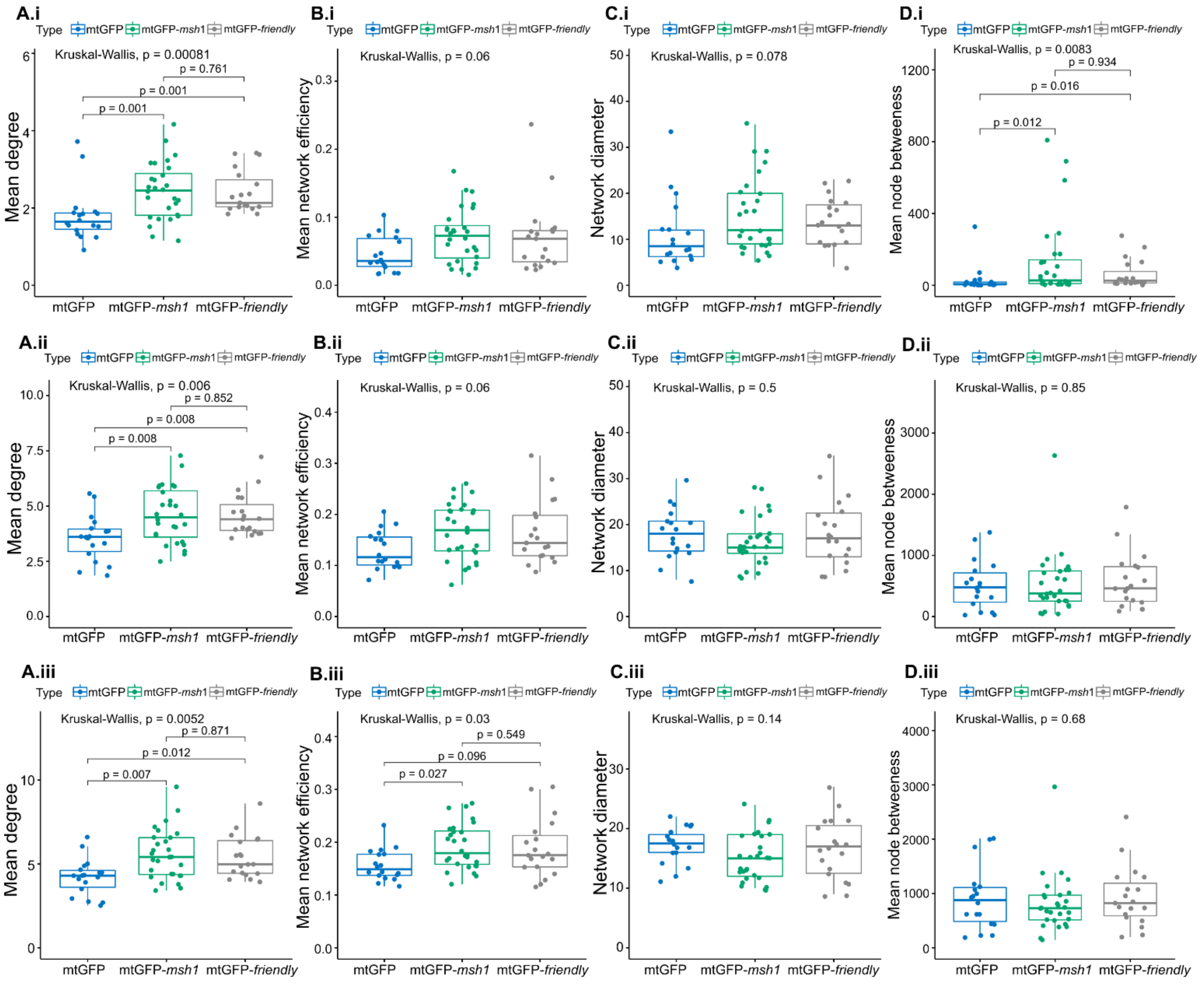

Supplementary Figure 8: Social summary statistics (A-D) provide evidence of differences between mtGFP, mtGFP-*msh1* and mtGFP-*friendly,* at three earlier time points (10 frames (i), 50 frames (ii), 100 frames (iii). Each point represents a summary statistic for one cell (mtGFP *n*= 18, mtGFP-*msh1 n*= 28, mtGFP-*friendly n* = 19*)*. P-values represent Kruskal-Wallis test outcomes across all three genotypes, and pairwise p-values are false discovery rare adjusted outcomes of a post-hoc Dunn test, without multiple hypothesis correction across statistics. Boxplots represent the median and 25th/75th percentile, with whiskers showing the smallest/largest value within 1.5x the interquartile range. Frames correspond to 19, 97 and 194 seconds, respectively. P-values are for individual experiments.

[available online]

Supplementary Video 1:

An example cell from 4-5 day old mtGFP-*msh1* hypocotyl, showing GFP-tagged mitochondria (green), and a Propidium Iodide stain around the cell (red); autofluorescence from the chloroplasts also detected (red).
